# Postnatal *Slc26a4* gene therapy improves hearing and structural integrity in a hereditary hearing loss model

**DOI:** 10.1172/JCI193812

**Published:** 2026-02-17

**Authors:** Yi-Hsiu Tsai, Peng-Yu Wu, Yu-Chi Chuang, Chun-Ying Huang, Hiroki Takeda, Hiroshi Hibino, Chen-Chi Wu, Yen-Fu Cheng

**Affiliations:** 1 Institute of Brain Science, College of Medicine, National Yang Ming Chiao Tung University, Taipei, Taiwan.; 2 Department of Medical Research, Taipei Veterans General Hospital, Taipei, Taiwan.; 3 Gene Therapy Research Center, National Yang Ming Chiao Tung University, Taipei, Taiwan.; 4 Division of Glocal Pharmacology, Department of Pharmacology, Graduate School of Medicine,; 5 Department of Otorhinolaryngology, Graduate School of Medicine, and; 6 Global Center for Medical Engineering and Informatics, The University of Osaka, Osaka, Japan.; 7 Department of Otolaryngology, National Taiwan University Hospital, Taipei, Taiwan.; 8 Department of Medical Research, National Taiwan University Hospital Hsin-Chu Branch, Hsin-Chu City, Taiwan.; 9 Department of Otolaryngology-Head and Neck Surgery, Taipei Veterans General Hospital, Taipei, Taiwan.

**Keywords:** Genetics, Otology, Gene therapy

## Abstract

Mutations in *SLC26A4* are the second most common cause of hereditary hearing loss (HL) in many Asian countries, leading to DFNB4, a condition characterized by progressive HL and inner ear malformations. While gene therapy holds great potential, its postnatal application has remained unexplored because of the lack of suitable animal models and the challenges of prenatal intervention. To our knowledge, this study represents the first preclinical investigation of postnatal gene therapy for DFNB4 using a clinically relevant *Slc26a4*-mutant mouse model that closely replicates human auditory phenotypes. Utilizing the synthetic AAV.Anc80L65 vector, we achieved robust *SLC26A4* delivery to critical cochlear regions, including the endolymphatic sac and cochlear lateral wall. Comprehensive phenotypic analyses revealed a critical therapeutic window spanning the neonatal and juvenile stages, within which AAV.Anc80L65-mediated *SLC26A4* delivery significantly improved hearing, as evidenced by lower auditory brainstem response thresholds. Moreover, the therapy preserved hair cells, reduced endolymphatic sac enlargement, partially restored the endocochlear potential, and mitigated inner ear structural degeneration. These therapeutic effects persisted into adulthood, highlighting the long-term efficacy of postnatal gene therapy. Together, these findings establish a critical therapeutic window for DFNB4 and demonstrate the feasibility of targeting the endolymphatic sac and cochlear lateral wall for effective intervention.

## Abstract 2

This study illustrates that postnatal gene therapy targeting the endolymphatic sac and spiral prominence region during a critical therapeutic window in the neonatal-juvenile period, employing a synthetic Anc80L65 vector, effectively enhances auditory function, maintains cochlear integrity, and confers enduring advantages in hereditary hearing loss caused by *SLC26A4* mutation.

## Graphical abstract

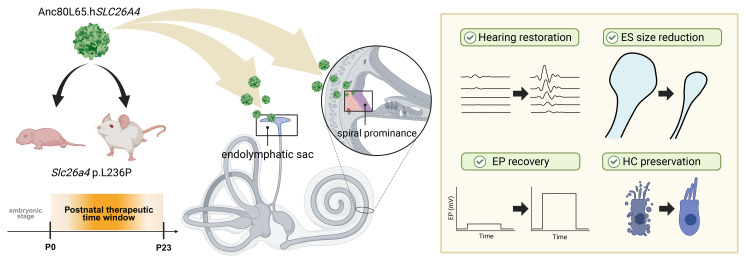

## Introduction

Hereditary hearing loss (HL) affects millions of people worldwide, with mutations in the *SLC26A4* gene recognized as a leading genetic cause, second only to *GJB2* mutations in many Asian countries (1–4). The *SLC26A4* gene encodes pendrin, an anion transporter critical for maintaining inner ear homeostasis. Pathogenic variants in the *SLC26A4* gene result in autosomal recessive disorders, including DFNB4 and Pendred syndrome (PDS), both characterized by a congenital, fluctuating, and severe-to-profound HL, with an enlarged vestibular aqueduct and endolymphatic sac (5, 6).

Pendrin mediates the transport of chloride (Cl^–^) and bicarbonate (HCO_3_^–^) ions between endolymph and perilymph, maintaining ionic balance in the inner ear. It is highly expressed in both the endolymphatic sac, where it regulates endolymph volume and composition, and the spiral prominence region of the cochlear lateral wall, where it overlaps with root cells and spindle-shaped cells of the stria vascularis (7–9). Dysfunction of pendrin disrupts inner ear fluid homeostasis, leading to fluid accumulation and enlargement of the endolymphatic sac and HL (10, 11). Recent studies have shown that targeting the endolymphatic sac for pendrin restoration can ameliorate cochlear hydrops and associated HL, underscoring its importance as a therapeutic target for DFNB4 (12).

Despite advances in understanding the molecular mechanisms underlying *SLC26A4-*related HL, effective therapeutic strategies remain limited. Previous gene replacement studies in *Slc26a4*-mutant mice, such as *Slc26a4*^Δ*/*Δ^ (or *PDS^–/–^* and *Slc26a4^–/–^* in some literature), *Slc26a4^loop/loop^* mice, and *Slc26a4^tm1Dontuh/tm1Dontuh^* mice demonstrated hearing restoration through prenatal (embryonic) delivery of *Slc26a4* cDNA, highlighting the potential of gene supplementation strategies (13, 14). However, these interventions were restricted to embryonic stages, partly due to the severe-to-profound HL and inner ear structural deformities seen in these animal models. Importantly, these models failed to replicate the phenotypes observed in patients with DFNB4, such as a residual hearing threshold after birth, thereby limiting their translational impact.

Recently, a p.L236P (L236P) mouse model harboring a c.T707C mutation in the *Slc26a4* gene, which results in a leucine (L) to proline (P) shift in amino acid levels, has provided a transformative platform for studying DFNB4 (15). Unlike other animal models, the L236P-mutant mice exhibit variable hearing phenotypes that closely resemble the heterogeneity observed in humans with DFNB4, making it a suitable platform for testing therapeutic interventions aimed at correcting the underlying defect.

Gene therapy has emerged as a promising strategy to treat hereditary HL by addressing its root genetic cause. Recent preclinical and clinical investigations have demonstrated that inner ear gene delivery can achieve significant hearing recovery in animal models of hereditary deafness (16–26). Adeno-associated virus (AAV) has emerged as a widely utilized viral vector for gene therapy (26–30), with demonstrated efficacy in targeting various inner ear cell types, including hair cells, supporting cells, and cells of the cochlear lateral wall (31–33). Nevertheless, achieving efficient transduction in the endolymphatic sac remains a challenge. Among existing serotypes, only WT AAV8 and synthetic AAV8BP2 have shown modest transduction efficiency in this critical structure (32). Given the pivotal role of the endolymphatic sac in DFNB4 pathophysiology, selecting AAV vectors with enhanced tropism and transduction efficiency for this organ is essential for effective gene therapy.

To our knowledge, this study represents the first investigation of postnatal *SLC26A4* gene supplementation therapy in a DFNB4 mouse model, targeting the optimal therapeutic window in the newborn and hearing-mature juvenile stages. We investigated the transduction efficiency of multiple AAV capsids in the endolymphatic sac and the spiral prominence region of the lateral wall. Among these, AAV.Anc80L65 demonstrated superior transduction efficiency and was subsequently utilized to deliver the *SLC26A4* transgene. Our results demonstrated marked improvements in auditory function and cochlear homeostasis, including reduced auditory brainstem response (ABR) thresholds and restoration of the endocochlear potential (EP), alongside the preservation of inner ear structural integrity, such as the maintenance of hair cell populations, stria vascularis morphology, and endolymphatic sac size. These findings underscore the feasibility of postnatal gene therapy targeting the endolymphatic sac and the spiral prominence as a comprehensive strategy for addressing both functional and structural deficits in DFNB4, filling a critical unmet need in the treatment of hereditary HL.

## Results

### Exploring the therapeutic window for gene therapy in a l236p dfnb4 mouse model

A critical challenge in the development of gene therapy for DFNB4 lies in identifying the optimal therapeutic window to prevent or reverse progressive HL, physiological dysfunction (such as reduced EP), and structural abnormalities. The *Slc26a4* L236P mouse model, which demonstrated early-onset moderate-to-profound HL (15), mimics the human phenotypes with variable hearing thresholds at birth. However, previous studies lacked long-term follow-up and comprehensive physiological characterization, limiting their utility in defining intervention timing and therapeutic efficacy. To address this, we conducted a comprehensive phenotypic characterization of L236P-mutant mice on a FVB/NJ Narl mice (FVB) background over an extended timeline (Supplemental Figure 1A; supplemental material available online with this article; https://doi.org/10.1172/JCI193812DS1).

#### HL progression and variability.

**Figure 1:**
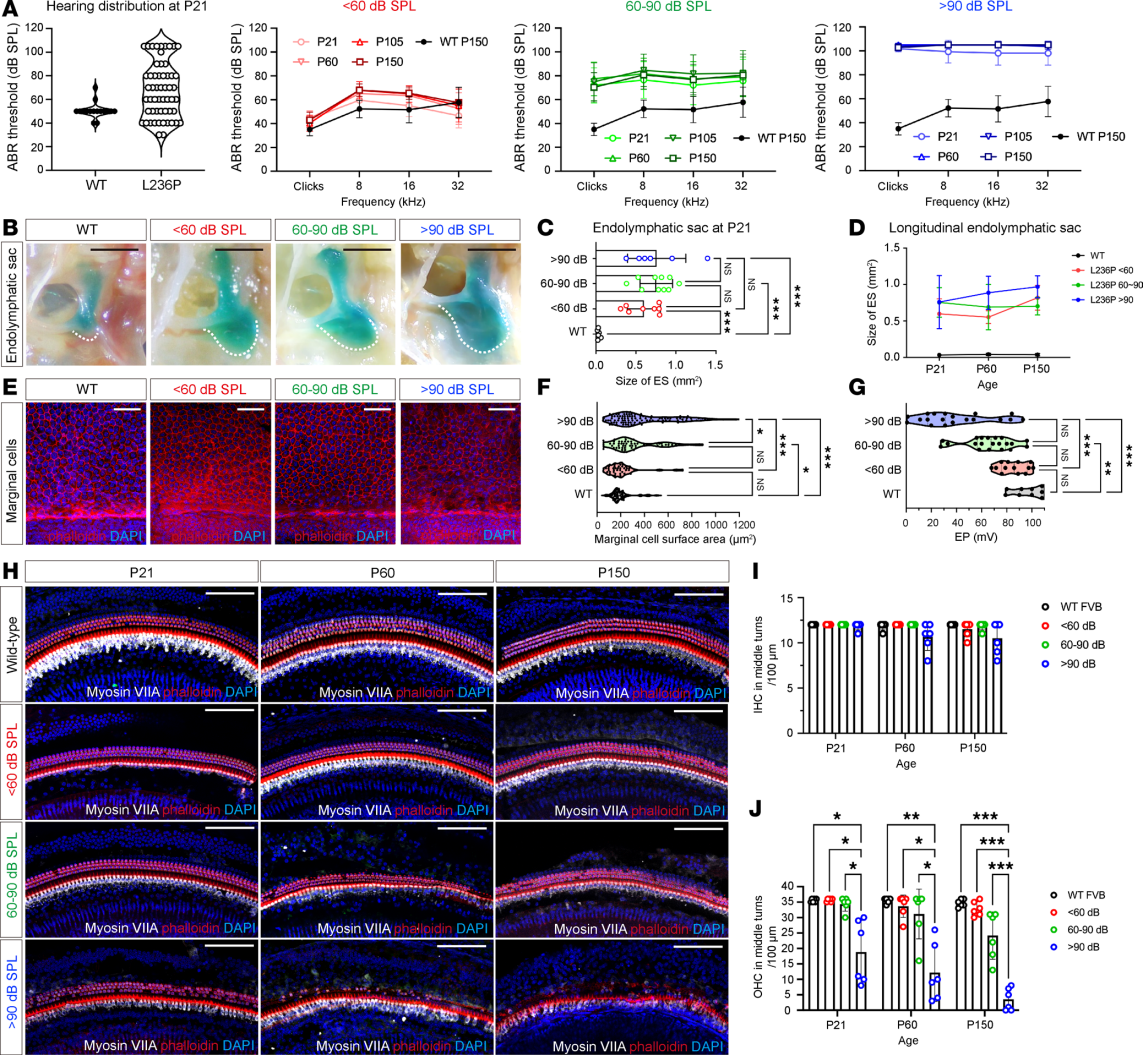
Phenotypic characterization of auditory function and cochlear anatomical anomalies in L236P-mutant mice. (**A**) L236P-mutant mice exhibited varying degrees of HL at P21, categorized into 3 groups: <60 dB (red, *n* = 23), 60–90 dB (green, *n* = 26), and >90 dB (blue, *n* = 10). WT mice served as controls (*n* = 16). (**B**) Representative images of endolymphatic sacs from WT and L236P mice across hearing groups at P21 (sacs are outlined by white dashed lines). Scale bars: 1 mm. (**C**) Quantification of the endolymphatic sac area at P21. Data are presented as the mean ± SD (*n* = 5–9 mice per group). ***P < 0.001, by 1-way ANOVA multiple comparisons with Tukey’s test). (**D**) Endolymphatic sac area at subsequent time points. Data represent the mean ± SD (*n* = 5–9 mice per group). (**E**) Representative images of marginal cells in the stria vascularis at P21. Scale bars: 50 μm. (**F**) Quantification of the surface area of marginal cells at P21. Data are presented as the mean ± SD (*n* = 45–105 cells per group). *P = 0.02 and ***P < 0.001, by 1-way ANOVA with Tukey’s test. (**G**) EP values for WT and L236P mice stratified by hearing thresholds: < 60 dB (*n* = 13); 60–90 dB (*n* = 20); and higher than 90 dB (*n* = 19). Data are presented as the mean ± SD. **P = 0.001 and *** *P* < 0.001, by Kruskal-Wallis test with Dunn’s test. (**H**) Hair cell morphology in the cochlear basal turn. Myosin VIIA (gray), phalloidin (red), and DAPI (blue) stainings are shown. Scale bars: 100 μm. (**I** and **J**) Quantification of basal IHCs and OHCs. Data are presented as the mean ± SD (*n* = 6). *P < 0.05, ** *P* < 0.01, and ***P < 0.001, by 2-way ANOVA with Dunnett’s test.

At P21, L236P-mutant mice exhibited varying degrees of HL (Figure 1A, hearing distribution at P21), similar to that in patients with DFNB4. To facilitate a detailed analysis and comparison, the mice were categorized into 3 groups on the basis of their ABR thresholds under 32 kHz stimulation: (a) mild HL (<60 dB SPL; Figure 1A, panel with red lines), (b) moderate HL (60–90 dB SPL; Figure 1A, panel with green lines), and (c) severe HL (>90 dB SPL; Figure 1A, panel with blue lines). This grouping allowed for the investigation of phenotypic variability and the relationship between hearing thresholds and structural changes. At P21, 22.73% (10 of 44 mice) exhibited mild HL, 56.81% (25 of 44 mice) moderate HL, and 20.45% (9 of 44 mice) severe HL. Notably, approximately 34.48% of the L236P mice had asymmetric HL, defined as an interaural difference of 20 dB or greater at 16 and 32 kHz. This asymmetry mirrored the clinical heterogeneity often observed in patients with DFNB4 (34, 35). Longitudinal assessment from P21 to P150 revealed that 75.86% (44 of 58 mice) maintained an ABR threshold within their initial category across multiple time points (P21, P60, P105, and P150), whereas some mice had fluctuations in their measured hearing (Supplemental Figure 1B).

### Correlation of hearing impairment with endolymphatic sac enlargement, marginal cell Atrophy, and EP

Several studies have shown that most mouse models of *Slc26a4* deficiency exhibit inner ear structural abnormalities, including an enlarged endolymphatic sac and vestibular aqueduct, marginal cells atrophy, abnormal EP, and hair cell loss (36–39). To investigate these changes in the L236P-mutant model, we examined the inner ear morphology at P21. Similar to other *Slc26a4*-deficient mouse models, all L236P-mutant mice displayed an enlarged endolymphatic sac at P21 (Figure 1B, white dashed lines). Mice with more severe hearing impairment tended to have a larger endolymphatic sac size (Figure 1B). Specifically, the average endolymphatic sac area in mice with severe HL (>90 dB) was 0.759 ± 0.364 mm² compared with 0.034 ± 0.016 mm² for WT FVB mice (*P* < 0.001). Similarly, mice with mild (<60 dB) and moderate (60–90 dB) HL had enlarged sac areas of 0.599 ± 0.206 mm² and 0.755 ± 0.203 mm², respectively (*P* < 0.001 vs. WT for both; *n* = 6–10 in each group). However, the differences in endolymphatic sac size among the mild, moderate, and severe HL groups were not statistically significant at P21 (Figure 1C) or at subsequent time points (Figure 1D). Furthermore, quantitative analysis of cochlear cross-sections revealed that, unlike other *Slc26a4*-deficient models, L236P mice did not exhibit significant scala media enlargement (Supplemental Figure 2).

Another common histopathological feature in the *Slc26a4*-deficient mouse model is the atrophy of marginal cells in the stria vascularis (38, 39), a structure essential for maintaining the ionic composition of the endolymph. To further evaluate the physiological effect of this abnormality, we quantified the surface area of the marginal cells in each hearing group of L236P mice (Figure 1, E and F). Compared with WT FVB mice (199.17 ± 84.40 μm^2^), L236P mice with mild, moderate, or severe HL exhibited average marginal cell areas of 207.72 ± 134.54 μm^2^ (*n* = 9), 314.09 ± 198.63 μm^2^ (*n* = 12) (vs. WT, *P* = 0.02), and 414.50 ± 288.91 μm^2^ (*n* = 21) (vs. WT, *P* < 0.001), respectively. Among these 3 hearing groups, mice with severe HL displayed significantly larger marginal cells than did mice in the mild (*P* < 0.001) and moderate (*P* = 0.02) HL groups (Figure 1F). Quantification of marginal cells at P150 revealed no significant difference in cell numbers between L236P and WT mice (Supplemental Figure 3), indicating that the observed pathology represented cellular atrophy rather than cell loss.

Corresponding differences were observed in EP values. EP measurements were assessed 1 day after ABR testing in L236P mice across the 3 hearing groups, using age-matched WT FVB mice as controls. Compared with WT mice, L236P mice exhibited a broad range of EP values (0.6–101.1 mV *n* = 52 mice) and a significantly reduced mean EP (WT: 98.97 ± 12.17 mV vs. L236P: 60.91 ± 27.99 mV, *P* < 0.001) (Supplemental Figure 4A). When stratified by hearing phenotype, EP values averaged 85.18 ± 10.98 mV, 65.73 ± 19.47 mV, and 39.23 ± 28.13 mV in the mild, moderate, and severe HL groups, respectively (Figure 1G). Spearman’s rank correlation analysis revealed a significant negative correlation between EP and the 32 kHz hearing threshold (*r* = –0.663, *P* < 0.001, *n* = 52) (Supplemental Figure 4B), indicating that EP deficits in L236P mice closely correlated with hearing dysfunction.

### Hair cell counts and potential therapeutic windows

To identify the optimal intervention time points for gene therapy, we first investigated cochlear structures at the neonatal stage (Supplemental Figure 5). Examination of hair cell numbers revealed that L236P-mutant mice maintained inner hair cell (IHC) and outer hair cell (OHC) counts comparable to those of WT mice at P5, regardless of their later auditory phenotypes, including the mild, moderate, and severe HL categories established on the basis of ABR thresholds. Quantitative analysis confirmed that there were no significant differences between L236P mutants and WT controls across any of the cochlear regions (Supplemental Figure 5B). These findings suggest that gene therapy intervention at the neonatal stage has the potential to prevent subsequent hair cell loss and associated hearing impairment in L236P-mutant mice.

We further examined the hair cell numbers and their correlations with hearing status in juvenile and adult mice. Whole-mount immunofluorescence staining of cochlear hair cells revealed no statistically significant differences in IHC counts between L236P-mutant mice and WT FVB mice across different cochlear turns, ages (from P21to P150), or hearing performance (Figure 1, H and I). However, OHC counts in the severe HL group were significantly lower at P21 compared with the moderate HL group (*P* = 0.03), the mild HL group (*P* = 0.04), and the WT group (*P* = 0.04), whereas no significant difference was seen among the other groups (Figure 1J, blue bar). The progressive OHC loss in the severe HL group was further observed at P60 and P150 (Figure 1, H and J) using 2-way ANOVA (*n* = 6 in each group and timing). Mice with moderate HL (Figure 1J, green bars) also showed progressive loss of basal OHCs at P150, while mice with mild HL maintained OHC numbers comparable to those in age-matched WT controls (Figure 1J).

Overall, our findings highlight 2 potential time points for gene therapy intervention in L236P mice. First, the neonatal stage (P5) appears to be a promising window for intervention, as hair cell counts remained intact at this age. Gene therapy administered at this stage could potentially prevent subsequent hair cell loss and hearing impairment. Second, the juvenile stage (P21) may be an effective intervention point, particularly for mice with moderate HL, as their IHCs and OHCs were largely intact. However, mice with severe HL at P21 already showed significant OHC loss, suggesting that earlier intervention, possibly at the neonatal stage, may be necessary for this group to maximize therapeutic benefit.

### AAV-mediated gene therapy in L236P mice

#### AAV.Anc80L65 demonstrates high transduction efficiency in the endolymphatic sac.

**Figure 2:**
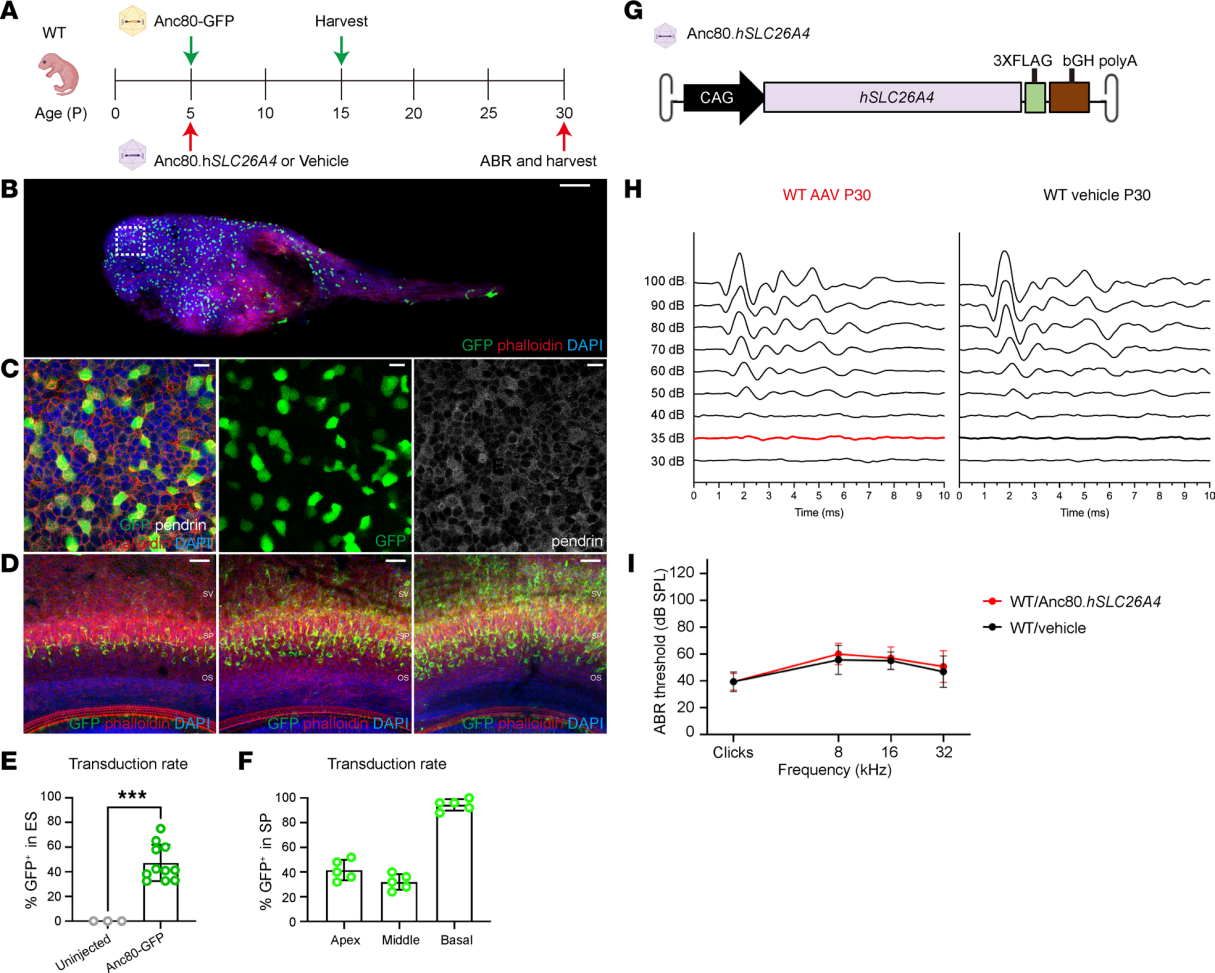
The AAV.Anc80L65 vector effectively transduces the endolymphatic sac and lateral wall cells without affecting hearing in WT mice. (**A**) Experimental timeline of neonatal delivery of Anc80.GFP and Anc80.*hSLC26A4* to WT mice. The illustration was created with BioRender.com. (**B** and **C**) The transduction tropism of Anc80.GFP in the endolymphatic sac of WT mice. Original magnification of confocal images, ×10 (**B**) and ×40 (**C**, zoomed-in white square area in **B**). The green, gray, red, and blue channels represent GFP, pendrin, phalloidin, and DAPI, respectively. Scale bars: 100 μm (**B**) and 10 μm (**C**). (**D**) Transduction tropism of Anc80.GFP in spiral prominence cells in the lateral wall of WT mice. The green, gray, red, and blue channels represent GFP, pendrin, phalloidin, and DAPI, respectively. Scale bars: 50 μm. (**E**) Quantification of the images in **C**. Data are presented as the mean ± SD (*n* = 11 AAV-injected mice). ***P < 0.001, by unpaired, 2-tailed *t* test. (**F**) Quantification of the images in **D**. Data are presented as the mean ± SD (*n* = 11 AAV injected mice). (**G**) Schematic diagram of the transgene construct. The full-length *hSLC26A4* coding sequence and FLAG sequence were driven by a CAG promoter (CMV enhancer and CBA promoter), followed by the bovine growth hormone (bGH) polyA sequence, flanked by AAV2 ITR, and packaged into an AAV.Anc80L65 capsid. (**H**) ABR waveforms of WT mice injected by Anc80.*hSLC26A4* (left panel) and vehicle (right panel) under 32 kHz stimulation at P30. The threshold is represented in bold lines in each panel. (**I**) Mouse ABR thresholds under click, 8, 16, and 32 kHz stimulations. Data are presented as the mean ± SD (*n* = 8 injected mice in each group).

To identify a suitable AAV capsid for DFNB4 gene therapy, we examined the transduction efficiency of 4 WT AAVs (AAV1, AAV2, AAV8, and AAV9) and 4 synthetic AAVs (AAV2.7m8, AAVie, AAV-PHP.eB, and AAV.Anc80L65) in the endolymphatic sac and cochlear lateral wall. Each capsid was packaged with a CAG promoter driving GFP and administered into the inner ear of WT FVB mice via round window membrane (RWM) microinjection during the neonatal phase (Figure 2A). Ten days after the AAV-GFP injection, whole-mount samples of the inner ear from the pups were collected to assess transduction efficiency in the critical tissue. GFP-positive cells within the endolymphatic sac were identified and quantified.

All tested capsids were able to transduce the endolymphatic sac (Supplemental Figure 6 and 7) and spiral prominence (Supplemental Figures 8 and 9), with AAV.Anc80L65 demonstrating the highest efficiency in both tissues. In the endolymphatic sac, AAV.Anc80L65 achieved transduction rates of 47.16% ± 14.77% relative to DAPI (*n* = 11) and 53.13% ± 7.47% specifically within pendrin-positive cells (*n* = 4) (Figure 2, B, C, and E). Similarly, in the spiral prominence, transduction efficiency reached 88.00% ± 7.48% relative to DAPI (*n* = 8) and 67.22% ± 5.93% within pendrin-positive cells (*n* = 6) (Figure 2, D–F). The mean transduction efficiencies for the other capsids in the endolymphatic sac were as follows: AAV1 (9.43% ± 1.91%, *n* = 4), AAV2 (11.44% ± 4.09%, *n* = 7), AAV8 (27.15% ± 4.44%, *n* = 4), AAV9 (11.11% ± 2.22%, *n* = 3), AAV2.7m8 (8.30% ± 1.36%, *n* = 3), AAV-ie (18.48% ± 3.11%, *n* = 4), and AAV-PHP.eB (10.81% ± 2.28%, *n* = 3). In the spiral prominence, the transduction efficiencies of the AAVs were as follows: AAV1 (20.80% ± 6.57%, *n* = 5), AAV2 (1.60% ± 2.19%, *n* = 5), AAV8 (21.60% ± 4.56%, *n* = 5), AAV9 (4.00% ± 2.83%, *n* = 5), AAV2.7m8 (13.60% ± 9.21%, *n* = 5), AAV-ie (46.40% ± 10.81%, *n* = 45), and AAV-PHP.eB (12.00% ± 4.00%, *n* = 5). On the basis of these results, we identified AAV.Anc80L65 as the most effective capsid for transducing the endolymphatic sac and spiral prominence and subsequently utilized it in additional animal studies.

#### Neonatal gene therapy improves hearing and mitigates inner ear structural defects.

To test the therapeutic potential of gene therapy in L236P mice, we designed an AAV vector carrying a human *SLC26A4* transgene and 3 FLAG tags, driven by a CAG promoter and packed in an AAV.Anc80L65 capsid (Figure 2G). This vector, abbreviated as Anc80.*hSLC26A4*, was first evaluated for safety by injecting it into the RWM of WT FVB mice during the neonatal period. The ABR threshold measured 25 days after surgery revealed no significant differences between Anc80.*hSLC26A4*-injected mice and vehicle-injected control mice (Figure 2, H and I). The findings indicated that Anc80.*hSLC26A4* delivery through the RWM effectively transduced cells in the inner ear, specifically the endolymphatic sac and spiral prominence, without causing hearing impairment.

**Figure 3:**
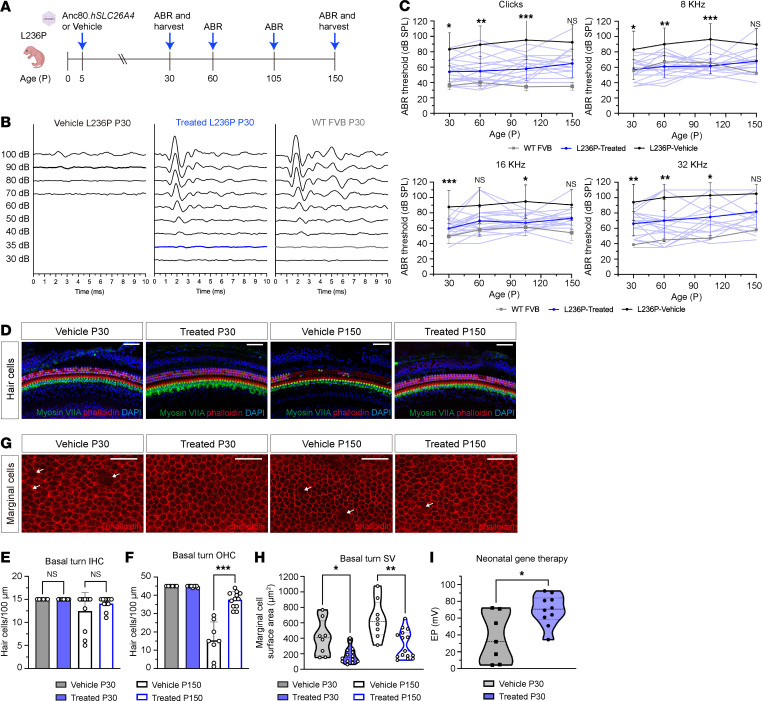
Neonatal delivery of Anc80.*hSLC26A4* improves hearing improvement and preserves cochlear structure in L236P-mutant mice. (**A**) Experimental timeline of neonatal delivery of Anc80.*hSLC26A4* to L236P-mutant mice. The illustration is created with BioRender.com. (**B**) Representative ABR waveforms for P30 L236P-mutant mice receiving a vehicle (left panel, threshold at 90 dB SPL in 32 kHz) or Anc80.*hSLC26A4* (middle panel, threshold at 35 dB SPL in 32 kHz) injection as neonates. Age-matched WT FVB mice were used as a control (right panel, threshold at 35 dB SPL in 32 kHz). (**C**) ABR thresholds of vehicle-treated (black lines) and Anc80.*hSLC26A4*-treated (dark blue lines; individual mice are represented by light blue lines) L236P-mutant mice. Age-matched WT FVB mice are represented by gray lines. Data are presented as the mean ± SD (*n* = 13–23 mice in each group). *P < 0.05, ** *P* < 0.01, and *** *P* < 0.001, by Kruskal-Wallis test with Dunn’s test. (**D**) Hair cell morphology at the cochlear basal turn in vehicle- and AAV-treated mice at P30 and P150. The green, red, and blue channels represent myosin VIIA, phalloidin, and DAPI, respectively. Scale bars: 100 μm. (**E** and **F**) Quantification of basal IHCs (**E**) and OHCs (**F**). Data are presented as mean ± SD. ***P = 0.004, by Mann-Whitney *U* test. (**G**) Morphology of marginal cells in the basal turn stria vascularis. Phalloidin staining (red) was used to delineate cell boundaries and epithelial organization. White arrows indicate atrophic marginal cells. Scale bars: 50 μm. (**H**) Quantification of the surface area of marginal cells. Data are presented as the mean ± SD. *P = 0.05 and ** *P* = 0.001, by ordinary 2-way ANOVA with Tukey’s multiple-comparison test. (**I**) EP value for vehicle-treated (*n* = 7) and AAV-treated L236P (*n* = 10) mice at P30. Data are presented as the mean ± SD. *P = 0.01, by unpaired, 2-tailed *t* test.

Our phenotyping analyses identified the neonatal stage (at least at P5) as a potential therapeutic window, during which gene therapy could potentially intervene to prevent or reverse inner ear abnormalities and, thus, the associated HL. To evaluate this hypothesis, Anc80.*hSLC26A4* vectors were administered through the RWM of newborn L236P-mutant mice, followed by audiological assessments to examine functionality and histological outcomes at P30, P60, P105, and P150 (Figure 3A).

Hearing ability was evaluated on the basis of ABR thresholds in response to click and pure tone (8, 16, and 32 kHz) sound stimuli. At the first measurement time point, P30, the AAV-injected L236P-mutant mice (Figure 3B, blue lines in right panel, threshold at 35 dB sound pressure level [SPL] in 32 kHz) exhibited improved hearing thresholds compared with the vehicle-injected littermates (Figure 3B, black lines in left panel, threshold at 90 dB SPL in 32 kHz). Lower ABR thresholds were observed in treated L236P-mutant mice at all the tested time points, from P30, P60, P105, to P150, across nearly all measured frequencies (Figure 3C) (vehicle, *n* = 13–21, vs. treated, *n* = 17–23, Kruskal-Wallis test with Dunn’s test). These findings indicated that neonatal injection of Anc80.*hSLC26A4* can improved hearing in L236P-mutant mice and that this effect was maintained for an extended duration.

Our previous findings indicated that L236P-mutant animals exhibited moderate-to-severe OHC loss, which resulted in hearing impairment. To assess whether neonatal gene therapy can prevent this hair cell loss, we examined histological alterations in the inner ear following Anc80.*hSLC26A4* delivery.

Cochlear tissues, including the organ of Corti and lateral wall, from AAV-treated and vehicle-injected L236P mice were collected at P30 and P150 and processed for whole-mount immunofluorescence staining (Figure 3, D and G). At P30, there were no significant differences in IHC or OHC counts between the 2 groups (Figure 3E), probably because most L236P mice still have regular hair cell numbers at this early age. However, at P150, the vehicle group showed a significant reduction in OHC numbers in the basal turn, whereas hair cell counts were maintained in the AAV-treated mice (Figure 3F; *P* < 0.001, Mann-Whitney *U* test, *n* = 8–12 in each group).

We also assessed the surface area of marginal cells in the stria vascularis using phalloidin staining (Figure 3, G and H). Neonatal injection of Anc80.*hSLC26A4* significantly mitigated marginal cell atrophy at both P30 (*P* = 0.01, Mann-Whitney *U* test, *n* = 15) and P150 (*P* = 0.004, Mann-Whitney *U* test, *n* = 15) compared with the vehicle-treated group (*n* = 8 at each time point). Consistent with the preservation in marginal cell morphology, we assessed the physiological function by measuring the EP at P30 (Figure 3I). Treated mice exhibited an average EP of 69.80 ± 18.04 mV (*n* = 10), which was significantly higher than that of the vehicle-treated mice (36.53 ± 29.48 mV, *n* = 7; *P* = 0.01, unpaired *t* test). These results indicate that neonatal gene therapy improved both the structural integrity and physiological function of the stria vascularis.

**Figure 4:**
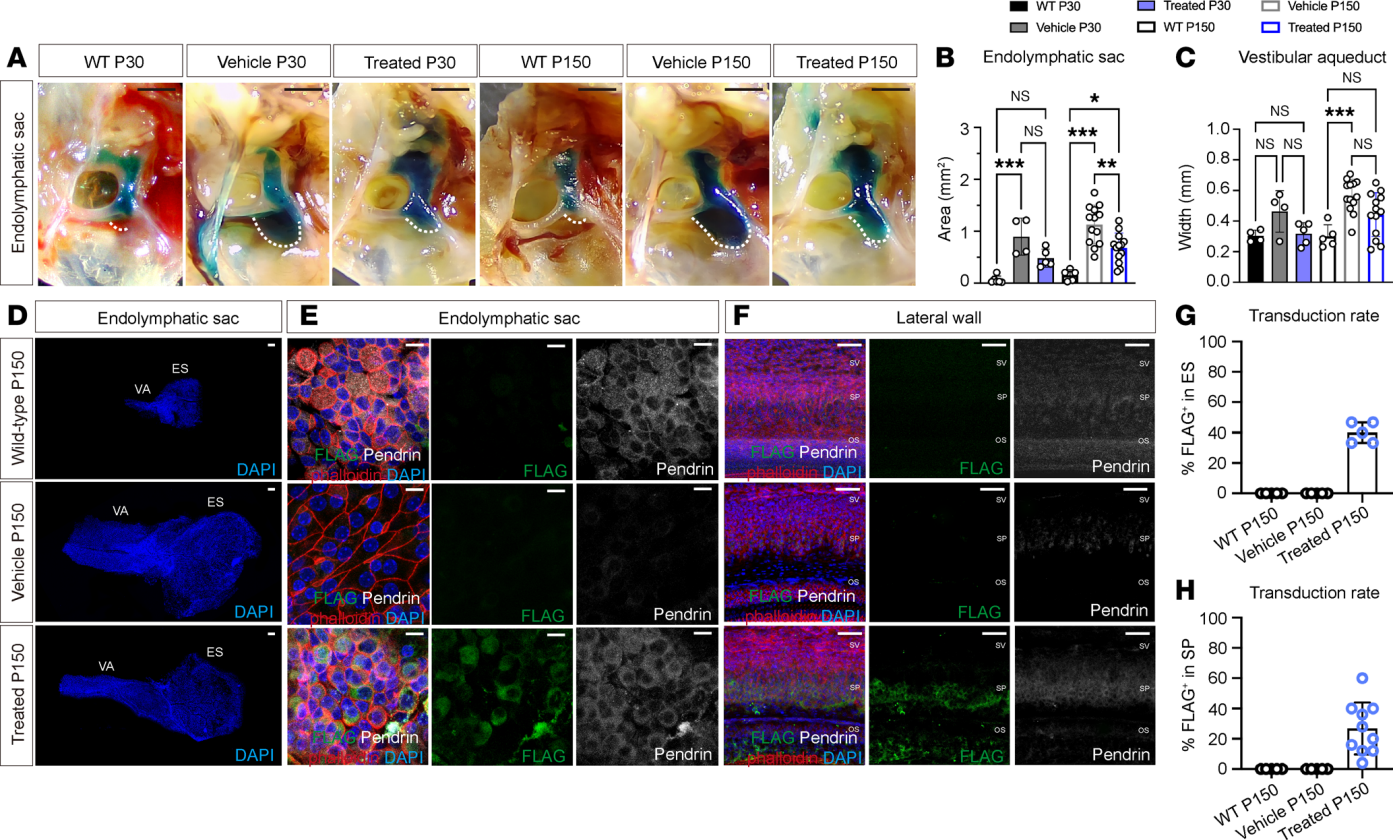
Neonatal delivery of Anc80.*hSLC26A4* reduces the enlarged endolymphatic sac in L236P-mutant mice. (**A**) Representative images of the endolymphatic sac of vehicle-treated and AAV-treated L236P-mutant mice at P30 and P150, compared with age-matched WT controls. Scale bars: 1 mm. (**B** and **C**) Quantification of the endolymphatic sac area (**B**) and vestibular aqueduct width (**C**) in vehicle-treated, AAV-treated, and age-matched WT mice. Data are expressed as the mean ± SD. *P = 0.01, **P < 0.03, and ***P < 0.001, by 1-way ANOVA with Tukey’s multiple-comparison test. (**D** and **E**) Confocal microscopy images showing the endolymphatic sac size (**D**) and transgene expression (**E**) in WT control, vehicle-injected, and AAV-treated L236P-mutant mice. The green, gray, red, and blue channels represent FLAG, pendrin, phalloidin, and DAPI, respectively. Scale bars: 100 μm (**D**) and 10 μm (**E**). (**F**) Confocal microscopy images showing transgene expression in the lateral wall in 3 groups of mice. The green, gray, red, and blue channels represent FLAG, pendrin, phalloidin, and DAPI, respectively. Scale bars: 50 μm. (**G**) Quantification of the images in **E**. Data are presented as the mean ± SD (*n* = 10 AAV-injected mice). (**H**) Quantification of the images in **F**. Data are presented as the mean ± SD (*n* = 10 AAV-injected mice).

L236P mice typically exhibit an enlarged endolymphatic sac and vestibular aqueduct from birth (Supplemental Figure 5A). We subsequently assessed whether neonatal gene therapy could ameliorate these structural anomalies. Compared with age-matched vehicle-injected mice, Anc80.*hSLC26A4*-treated mice exhibited a significant reduction in the endolymphatic sac size at P150 (Figure 4, A and B, vehicle vs. AAV-treated = 1.13 ± 0.37 vs. 0.68 ± 0.29 mm², *n* = 12, *P* = 0.03, Kruskal-Wallis test with Dunn’s multiple-comparison test), although the difference was not significant at P30 (vehicle vs. AAV-treated = 0.90 ± 0.36 vs. 0.48 ± 0.17 mm², *n* = 4–5, *P* = 0.71, Kruskal-Wallis test with Dunn’s multiple-comparison test). The width of the vestibular aqueduct showed reduced, yet no statistically significant, difference in the AAV-treated L236P-mutant mice (Figure 4, A and C; P30 vehicle vs. AAV-treated: 0.46 ± 0.14 mm vs. 0.32 ± 0.07 mm, *n* = 12, *P* = 0.8; P150 vehicle vs. AAV-treated: 0.57 ± 0.11 mm vs. 0.44 ± 0.14 mm, *n* = 14, *P* = 0.15).

Immunofluorescence staining at P150 confirmed the presence of FLAG-tagged pendrin in the endolymphatic sac of treated animals but not in vehicle-treated animals or WT controls (Figure 4, D, E, and G). Similar staining results were observed in the spiral prominence of the lateral wall (Figure 4, F and H), indicating successful transduction and expression of the *SLC26A4* transgene. The reduced endolymphatic sac size in treated mice suggests that overexpression of the functional *SLC26A4* transgene can rectify this structural defect.

#### Juvenile gene therapy improves hearing and mitigates cochlear structural abnormalities.

To examine the therapeutic potential of Anc80.*hSLC26A4* following the onset of hearing, we conducted gene therapy in juvenile L236P-mutant mice. Given the variability in hearing abilities and histological changes in this mouse model, we selected individuals with moderate HL (60–90 dB SPL threshold) at P21, characterized by the number of intact hair cells at P21, for further treatment. We first evaluated the transduction efficiency of *Anc80.hSLC26A4* in juvenile WT mice. The vector achieved transduction rates of 33.5% ± 17.85% in the endolymphatic sac (*n* = 8; Supplemental Figure 10, A, B, and D) and 23.00% ± 4.77% in the spiral prominence (*n* = 6; Supplemental Figure 10, C and E). These results confirm that Anc80 capsid successfully transduced the target regions even at the juvenile stage.

**Figure 5:**
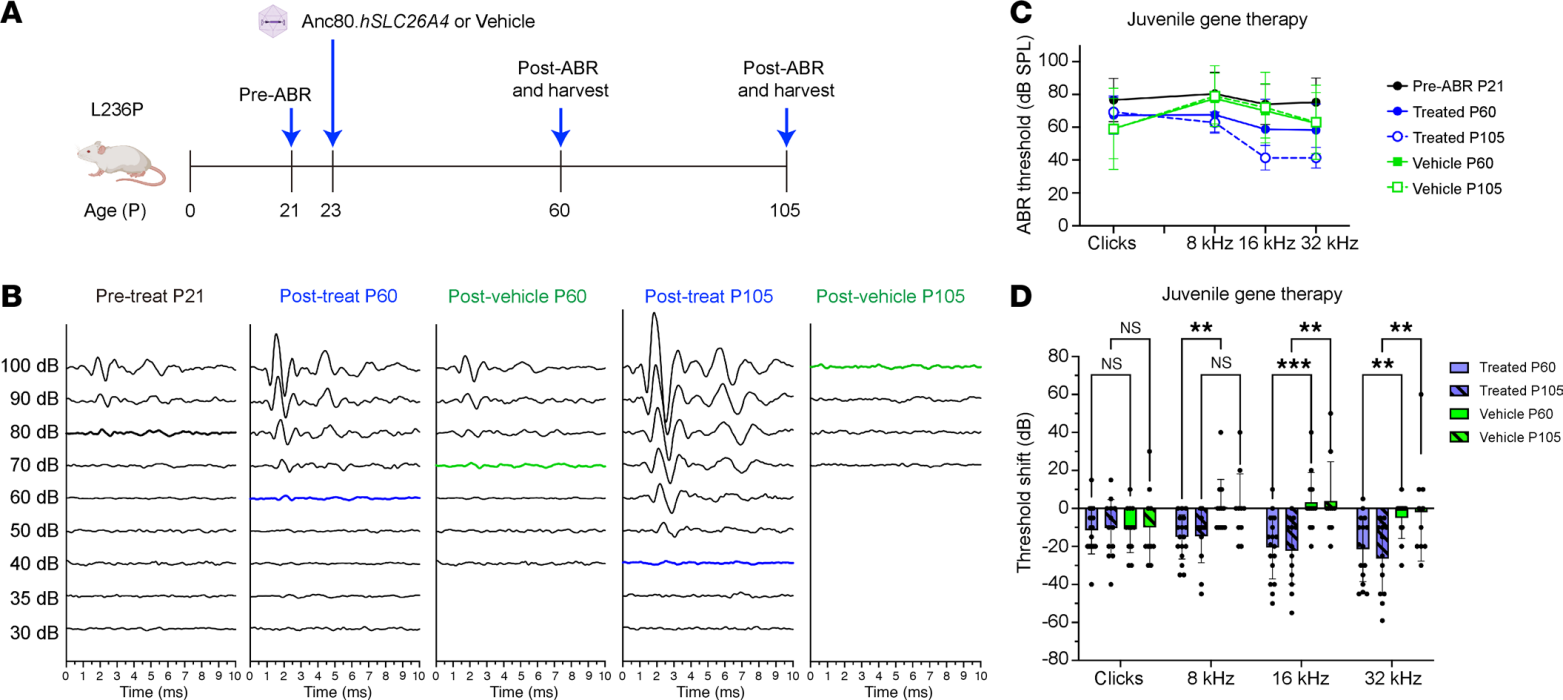
Delivery of Anc80.*hSLC26A4* improves hearing in L236P-mutant juvenile mice. (**A**) Experimental timeline of injection of Anc80.*hSLC26A4* into juvenile L236P-mutant mice, with pre-ABR measurements at P21 and post-ABR assessments at P60 and P105. The illustration was created with BioRender.com. (**B**) Representative 32 kHz ABR waveforms in L236P-mutant mice showing the pre-ABR (Pre-treat) at P21, the post-ABR (Post-treat) of AAV-treated animals at P60 and P105, and the post-ABR (Post-vehicle) of vehicle-injected animals at P60 and P105. (**C**) ABR thresholds for pre-ABR (black) and post-ABR in Anc80.*hSLC26A4*-treated (blue lines), vehicle-injected mice. Solid lines represent the threshold at P60; dashed lines represent the threshold at P105. (**D**) ABR threshold shifts calculated from **C**. Blue and green bars represent treated and vehicle-injected animals, respectively. Bars without and with slash lines represent post-ABR at P60 and P105, respectively. Negative values indicate hearing improvement. Data are presented as the mean ± SD. **P < 0.01 and *** *P* < 0.001, by 2-way ANOVA.

Before the treatment, mice underwent a pretreatment ABR (pre-ABR) test at P21 to establish their initial hearing threshold. Mice with an ABR threshold in the 60–90 dB SPL range were then randomly assigned to either the vehicle control or gene therapy group. Gene therapy was delivered via Anc80.*hSLC26A4* injection at P23. Post-treatment ABR (post-ABR) was measured at P60 and P105 (Figure 5A).

At P60, AAV-treated mice demonstrated substantial hearing improvements, with thresholds reduced to 60 dB SPL for 32 kHz stimuli (Figure 5B, blue lines in middle panel). Hearing thresholds in the AAV-treated group showed further improvement by P105 (Figure 5C).

To quantify the changes, we calculated the threshold shift (post-ABR minus pre-ABR values) ​​for each mouse, with negative values ​​indicating hearing improvement. At P60, AAV-treated mice showed significant hearing improvements compared with the control group, particularly at 8, 16, and 32 kHz. By P105, significant improvement persisted at 16 and 32 kHz frequencies (Figure 5D).

**Figure 6:**
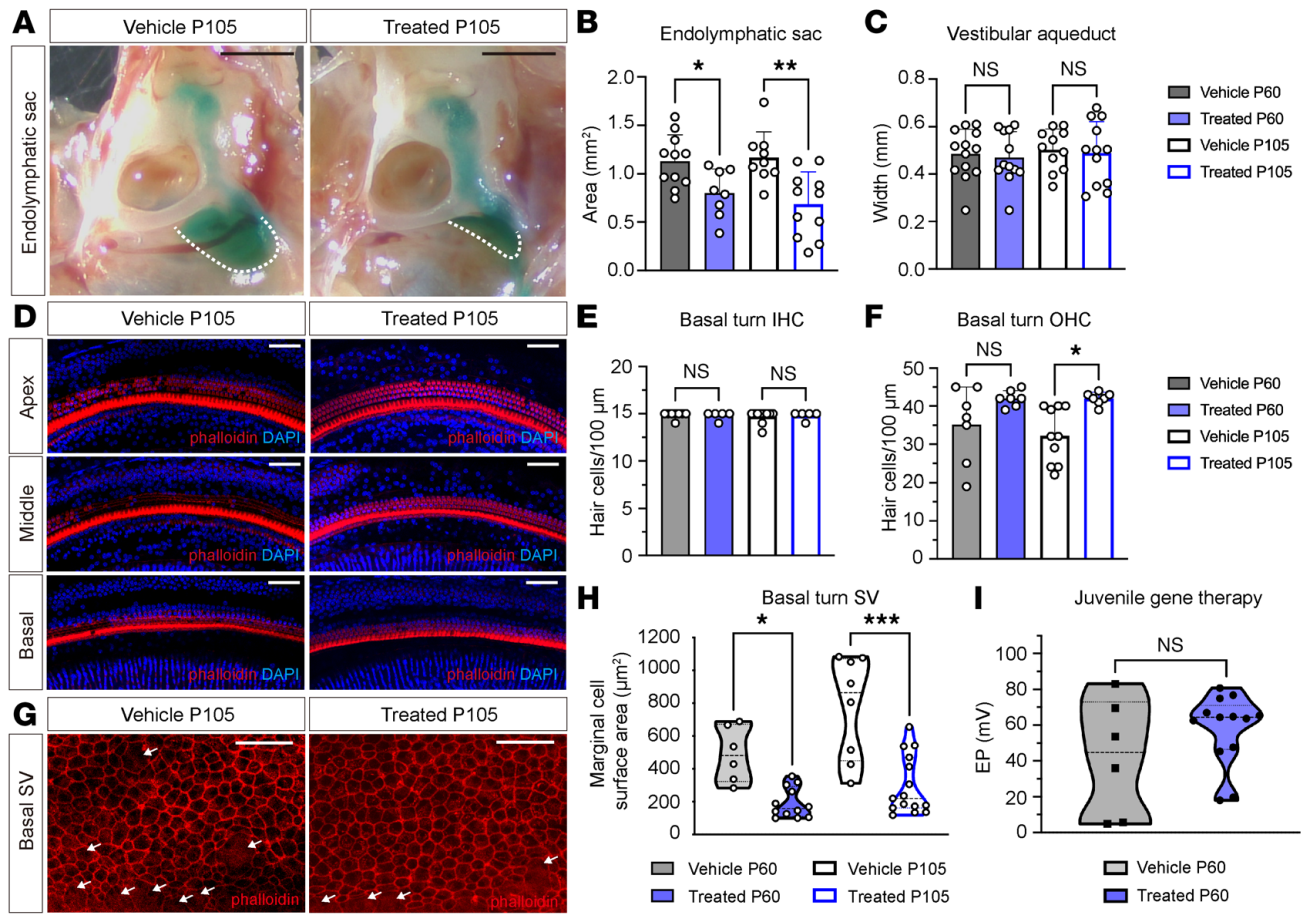
Juvenile delivery of Anc80.*hSLC26A4* reduces the size of the enlarged endolymphatic sac and improves cochlear structures in L236P-mutant mice. (**A**) Representative images of the endolymphatic sac of vehicle-injected and Anc80.hSLC26A4-treated L236P-mutant mice at P105. Scale bars: 1 mm. (**B** and **C**) Quantification of the endolymphatic sac area (**B**) and vestibular aqueduct width (**C**) at P60 and P105. Data are expressed as the mean ± SD. *P = 0.01 and **P = 0.002, by unpaired, 2-tailed *t* test. (**D**) Hair cell morphology in cochlear turns of vehicle-injected and Anc80.*hSLC26A4*-treated mice at P105. Red and blue channels represent phalloidin and DAPI, respectively. Scale bars: 100 μm. (**E** and **F**) Quantification of basal IHCs (**E**) and OHCs (**F**) at P60 and P105. Data are presented as the mean ± SD. *P = 0.02, by Kruskal-Wallis test. (**G**) Morphology of marginal cells in the basal turn stria vascularis. Phalloidin staining (red) was used to delineate cell boundaries and epithelial organization. White arrows indicate atrophic marginal cells. Scale bars: 50 μm. (**H**) Quantification of the surface area of marginal cells at P60 and P105. Data are expressed as the mean ± SD. *P = 0.02 and ***P = 0.01, by Kruskal-Wallis test with Dunn’s multiple-comparison test. (**I**) EP value for vehicle-treated mice (*n* = 6) and AAV-treated L236P mice (*n* = 13) at P60. Data are presented as the mean ± SD. Statistical significance was assessed by unpaired, 2-tailed *t* test.

Following the hearing assessment, L236P-mutant mice that underwent juvenile-stage gene therapy were analyzed at P60 and P105. At P105, AAV-treated mice exhibited a significant reduction in size in endolymphatic sac size compared with age-matched controls (Figure 6, A and B, P105 vehicle vs. AAV-treated = 1.17 ± 0.27 vs. 0.69 ± 0.33 mm², *n* = 11, *P* = 0.02, Kruskal-Wallis test). No significant differences in endolymphatic sac size were observed at P60, and measurements of the vestibular aqueduct width revealed no significant differences at either time point (Figure 6, B and C).

The number of hair cells and the surface area of marginal cells in the stria vascularis were determined following immunofluorescence labeling. At P105, AAV-treated L236P-mutant mice exhibited significantly higher OHC survival rates compared with control mice (Figure 6, D and F) and a normalized marginal cell area at both P60 and P105 (Figure 6, G and H). In contrast, no significant differences were observed in IHC counts at P60 and P105 Figure 6E), or in OHC counts at P60, likely due to the moderate baseline phenotype of the mice selected for juvenile intervention. Although marginal cell morphology was better preserved in the treated mice than in the vehicle-treated controls, the EP of juvenile-treated mice (55.59 ± 17.14 mV) was only moderately higher and showed only a modest, nonsignificant increase compared with the vehicle-treated group (42.10 ± 32.63 mV) (Figure 6I).

The juvenile-stage delivery of Anc80.*hSLC26A4* injections resulted in a reduced hearing threshold as early as P60, with further improvement observed at P105. This auditory enhancement was more pronounced and was accompanied by a reduction in endolymphatic sac size, prevention of OHC loss, and normalization of the stria vascularis structure. Together, these findings highlight the ability of juvenile gene therapy to mitigate cochlear structural alterations and improve auditory outcomes in L236P-mutant mice.

## Discussion

This study presents the successful application of postnatal gene therapy for DFNB4 in a clinically relevant mouse model, marking what we believe to be a groundbreaking approach to translating genetic therapies into clinical practice. Using the L236P mouse model, which recapitulates key features of the human DFNB4 phenotypes, including an enlarged endolymphatic sac and varying degrees of auditory abilities ranging from mild HL to total deafness, this research, in our view, highlights a significant advancement in the field. Our findings revealed significant correlations among hearing threshold, the presence of surviving hair cells, and the size of the endolymphatic sac, leading to the identification of a therapeutic time window for gene therapy intervention in L236P mice.

By administrating the therapeutic AAV vector Anc80.*hSLC26A4* at either neonatal (P5) or juvenile (P21) stages, we demonstrated sustained improvements in auditory outcomes, including preserved hearing thresholds and structural integrity by maintaining hair cell numbers and mitigating endolymphatic sac enlargement over time. This work establishes postnatal gene therapy as a feasible and effective strategy for DFNB4, addressing a critical unmet need and paving the way for translational applications in hereditary HL.

Central to the translational applicability of our research is the use of the *Slc26a4* p.L236P–knockin mouse, which carries a prevalent DFNB4 mutation found in humans (40). Previous *Slc26a4*-deficient mouse models — including *Slc26a4*^Δ*/*Δ^ (also referred as *Slc26a4^–/–^* or *Pds^–/–^* in some research) (41) and *Slc26a4^tm1Dontuh/tm1Dontuh^* (37) — often exhibit uniform, severe phenotypes (profound congenital deafness with gross inner ear malformations) that do not reflect the spectrum of human disease. For instance, *Slc26a4*-null mice are born profoundly deaf with enlarged and degenerative inner ear structures, an extreme outcome rarely seen in patients (36, 41). In contrast, the L236P model more closely mirrors the human condition: affected mice displayed variable HL (ranging from mild to profound). This phenotypic variability — along with only partial malformations such as an enlarged endolymphatic sac — aligns with DFNB4 presentations in humans. To capture the phenotypic heterogeneity typical of human *SLC26A4*–related deafness, we stratified L236P mice into mild (<60 dB), moderate (60–90 dB), and severe (>90 dB) HL categories. Unlike *Slc26a4*-KO mice, which consistently exhibit profound, early-onset HL (>90 dB), the variable expressivity of the L236P model uniquely enables the definition of therapeutic windows across a broad spectrum of disease severity. Importantly, this heterogeneity appears intrinsic to the p.L236P mutation rather than the FVB genetic background, as similar phenotypic variability was observed in L236P-mutant mice generated on a CBA/CaJ background. By recapitulating the nuanced human phenotype, the L236P model provides a translationally relevant platform to evaluate therapies under conditions that simulate the clinical scenario, enabling a more realistic evaluation of therapeutic interventions and increasing the translational relevance of our results.

Notably, while our L236P model demonstrates selective OHC vulnerability with relative preservation of IHCs, validating this pattern in human PDS remains challenging. Human temporal bone studies are rare and typically capture advanced stages of disease, in which widespread degeneration obscures the primary cellular pathology (12, 36, 42–44). Consequently, this mouse model provides critical insight into the early pathogenic sequence — specifically OHC susceptibility — that is difficult to capture in clinical specimens.

Our phenotypic analysis further elucidates the multifaceted pathology underlying auditory dysfunction in the L236P model. While OHC loss correlates with severe hearing thresholds, animals with mild-to-moderate HL exhibited significant reductions in EP and marginal cell atrophy despite relative OHC preservation. This indicates that HL in these groups was not driven solely by sensory cell death, but rather by the disruption of cochlear fluid homeostasis. Consequently, the therapeutic efficacy of postnatal gene therapy likely stemmed from the stabilization of the cochlear electrochemical environment, evidenced by the partial restoration of EP and stria morphology, which subsequently mitigated secondary sensory cell vulnerability.

In designing our juvenile intervention strategy, we specifically targeted the moderate HL subgroup to maximize the sensitivity of our therapeutic evaluation. The mild HL subgroup exhibited minimal hair cell loss at this stage, presenting a potential “ceiling effect” that could obscure the detection of therapeutic efficacy. Conversely, the severe HL subgroup displayed profound, likely irreversible, OHC degeneration by P21, rendering functional rescue unlikely. Thus, the moderate HL phenotype represents the optimal intermediate stage at which assess the potential for reversing established pathology. Future studies extending this intervention to mild and severe HL cohorts will be valuable for delineating the precise boundaries of the therapeutic window.

The developmental biology of the inner ear has long suggested that intervention must occur very early to prevent irreversible damage. Studies in mouse models pinpointed a critical window in late embryogenesis when pendrin is required for normal auditory development (45). Notably, Choi et al. demonstrated that restoring pendrin expression only from E16.5 to P2 was sufficient for mice to develop normal hearing (36). The absence of pendrin during this narrow time period led to endolymphatic abnormalities and HL — even without overt cochlear malformation. These insights raised an important issue about whether gene therapy should be delivered embryonically (in utero) to effectively prevent congenital HL. Kim et al. delivered therapeutic rAAV2/1-*Slc26a4* to *Slc26a4*^Δ*/*Δ^ and *Slc26a4^tm1Dontuh/tm1Dontuh^* mice at E12.5, resulting in a restoration of various hearing thresholds at 5 weeks of age (14). Takeda et al. used electroporation-mediated transuterine gene transfer to deliver *Slc26a4* cDNA into *Slc26a4^–/–^* mice at E11.5, achieving reductions in cochlear hydrops, improvements in balance function, and a 15–20 dB hearing threshold improvement at P30 (13). Indeed, both proof-of-concept studies, by delivering *Slc26a4* cDNA into embryonic mouse otocysts, successfully enabled the acquisition or improvement of hearing in pendrin-deficient mice. However, that embryonic gene delivery, while preventing inner ear enlargement and permitting hearing development, had notable shortcomings: it failed to establish stable long-term hearing. More important, translating an embryonic intervention to humans presents tremendous challenges. Embryonic gene therapy also carries unique ethical and practical concerns not encountered with postnatal treatment (46). These considerations underscore the fact that, despite the theoretical advantages of correcting inner ear defects before birth (47), embryonic gene therapy is fraught with feasibility issues that impede its clinical adoption. Consequently, successful intervention in the postnatal period represents a crucial step forward in the practical implementation of gene therapies for DFNB4.

Previous studies of *SLC26A4* gene therapy have highlighted the essential role of pendrin expression in the endolymphatic sac to restore cochlear function (12, 14). Additionally, pendrin expression in the spiral prominence region of the cochlear lateral wall is increasingly recognized as important: mice retaining partial pendrin expression (approximately 10% of WT levels) in the spiral prominence show slower progression of HL and reduced auditory fluctuations compared with animals completely lacking pendrin in this region, underscoring its significance in maintaining cochlear fluid homeostasis and auditory stability (8, 14). However, the efficacy of viral vectors in efficiently transducing the spiral prominence and endolymphatic sac has not been thoroughly characterized.

In this study, we evaluated the transduction efficiency of 8 different AAV vectors — 4 WT (AAV1, AAV2, AAV6, and AAV8) and 4 synthetic variants (AAV2.7m8, AAV8BP2, AAV9-PHP.B, and Anc80L65) — in the endolymphatic sac following neonatal RWM injection. Among these, the synthetic vector AAV.Anc80L65 achieved the highest transduction efficiency (~47%), markedly surpassing the efficiencies (~20%) previously reported with alternative vectors such as AAV8 and AAV8BP2 (32, 48). Given its superior transduction efficacy in the inner ear, particularly in the endolymphatic sac and spiral prominence, we chose Anc80L65 as the therapeutic vector to ensure broader functional restoration that would potentially improve therapeutic outcomes for DFNB4-associated HL.

Our findings indicate that postnatal pendrin overexpression via AAV delivery has the potential to improve auditory functions in *Slc26a4*-mutant mice. However, achieving full restoration of inner ear functions remains challenging, particularly in more mature animals. While juvenile gene therapy conferred significant auditory benefits, the magnitude of rescue was less robust when compared with neonatal intervention. Our analysis revealed markedly reduced transduction efficiency in the endolymphatic sac and spiral prominence of juvenile mice compared with neonates. This age-dependent reduction likely stems from structural maturation barriers and downregulation of the universal AAV receptor (AAVR), which has been reported to decline in the mature cochlea and limit AAV infectivity (49). Consequently, current vectors, including Anc80L65, may not fully replicate the physiological pendrin expression patterns — specifically in duration or distribution — required for more effective rescue. Future research should prioritize the development of next-generation AAV vectors or optimized constructs to enhance transgene expression efficiency and durability, particularly in the endolymphatic sac and cochlear lateral wall. These advancements would expand the applicability of gene therapy not only for DFNB4 but potentially for a wide range of hereditary hearing impairments.

## Methods

### Sex as a biological variable.

Our study examined male and female animals equally, and similar findings are reported for both sexes.

### Animals.

The *Slc26a4* L236P mouse model was created using the CRISPR/Cas9 genome-editing technique according to the design by Wen et al. (15) and maintained on an FVB/NJNarl background. The mice were supplied by the National Laboratory Animal Center (NLAC), NAR Labs in Taiwan. Overt vestibular dysfunction, characterized by circling behavior, was observed in less than 10% of the L236P mutant colony. To minimize phenotypic variability and focus on auditory outcomes, mice displaying circling behavior were excluded from the study.

### AAV.

The human *SLC26A4* cDNA sequence was verified before packaging into the AAV.Anc80L65 (Anc80) capsid derived by CAG ubiquitous promoter (composed of the CMV early enhancer, the chicken β-actin promoter, and the rabbit β-globin splice acceptor site). The Anc80.*hSLC26A4* (1.1 × 10^13^ gc/mL) and Anc80.GFP (1.0 × 10^13^ gc/mL) vectors were produced at SABTech by triple-plasmid transfection of adherent HEK293T cells followed by standard purification. Titers were confirmed using droplet digital PCR (ddPCR). Additional AAV serotypes, including AAV1 (1.0 × 10^13^ gc/mL), AAV2 (2.6 × 10^13^ gc/mL), AAV8 (1.0 × 10^13^ gc/mL), AAV9 (1.0 × 10^13^ gc/mL), AAV2.7m8 (1.0 × 10^13^ gc/mL), AAVie (1.0 × 10^13^ gc/mL), and AAV.PHP.eB (1.8 × 10^13^ gc/mL), carrying the GFP gene, were produced at the National RNAi Core Facility (Academia Sinica). For these, HEK293T cells were plated in 150 mm dishes and transfected the following day with transfer plasmid, helper plasmid (Takara), and a rep-cap plasmid using PEI-MAX (Polysciences) as the transfection agent. After transfection, the AAV-containing medium was collected and precipitated using a PEG-8000 mixture, followed by centrifugation at 3,200*g*. The resulting pellet was resuspended in an AAV buffer. After ultracentrifugation, AAV particles were isolated from the 40%–60% iodixanol interface using an 18 gauge needle and sterilized via a 0.45 μm syringe filter (Pall Corporation). The purified AAV particles were then concentrated using columns (EMD Millipore) and quantified by ddPCR.

### Surgical methods.

Neonatal surgery was performed at P5. Following hypothermic anesthesia, a post-auricular incision was made to reveal the RWM. A total of 1,000 nL AAV vector was injected into the inner ear at a controlled rate of 200 nL/min via glass micropipettes mounted on a Nanoliter Microinjection System (WPI). The pups were then placed under a 42°C heating light to aid in their rehabilitation.

Juvenile surgery was performed at P23, following the procedure described by Yoshimura et al. (50). Animals were anesthetized with an i.p. injection of Zoletil and Rompun. Once anesthetized, the mice were positioned laterally to expose the left side. A post-auricular incision was made, and the underlying muscles were removed to expose the bulla and posterior semicircular canal. A small hole was created in the posterior semicircular canal using tweezers, and the flow of perilymph confirmed successful penetration. The AAV vector was injected through the RWM using a 36 gauge NanoFil blunt iron needle (WPI) mounted on a Nanoliter Microinjection System (WPI). After the injection, the mouse was placed under a 42°C heating light to aid in recovery.

### Decalcification of cochlear tissue.

Cochlear tissues were harvested at P21, P105, and P150. Following fixation with 4% paraformaldehyde (PFA), the tissues underwent immersion in 0.5 M EDTA for decalcification. The decalcification solution was discarded the next day, and the fresh solution was refilled. After 5 days or more of this process, the tissues attained the desired level of softness for further experimentation.

### Quantification of endolymphatic sac size and vestibular aqueduct width.

Following decalcification, the endolymphatic sac and vestibular aqueduct were visualized after being filled with fast green dye. The dye was injected into the observed tissue using glass micropipettes on a Nanoliter Microinjection System (WPI).

### Immunofluorescence staining.

Cochlear tissues were harvested at P21, P60, P105, and P150. After collection, the tissues were decalcified in a solution containing 4% PFA and EDTA. The dissected samples were permeabilized with 1% Triton X-100, thoroughly washed with PBS, and subsequently blocked with PBT1 (1% BSA and 0.5% Triton X-100) solution. The samples were then incubated with the appropriate primary antibodies and secondary antibodies, including anti–myosin VIIA (25-6790, Proteus Biosciences), anti-FLAG (8146, Cell Signaling Technology), customer-made anti-pendrin as previously reported (3), anti–rabbit Alexa Fluor 633 (SAB4600132, MilliporeSigma), and anti–mouse Alexa Fluor 488 (A-21202, Invitrogen, Thermo Fisher Scientific).

After removing unbound antibodies, the samples were stained with phalloidin and DAPI at room temperature. Finally, the samples were mounted with a fluorescent mounting medium and imaged using confocal microscopy (Zeiss LSM 880).

### Quantification of marginal cell surface area.

The quantification method used was adapted from Jabba et al. (39). Marginal cell boundaries in the cochlear stria vascularis were visualized using phalloidin labeling and imaged by confocal microscopy. For each image of the stria vascularis, 5 regions of 2,500 μm² were randomly selected. The surface area of individual marginal cells within these regions was manually quantified using ImageJ (NIH) by tracing the phalloidin-defined cell boundaries.

### Quantification of scala media enlargement.

Assessment of cochlear hydrops followed the method described by Johns et al. (51). The distension of Reissner’s membrane was measured to quantify the enlargement of scala media. Using ImageJ, the actual path length of Reissner’s membrane was traced and compared with the linear distance between its 2 attachment points (defined as the idealized reference length). The ratio of the actual length to the idealized length was calculated, with higher ratios indicating a greater degree of scala media enlargement.

### ABR analysis.

ABR measurements were conducted on anesthetized mice using sound stimuli at clicks, 8 kHz, 16 kHz, and 32 kHz, with data acquired via BiosigRZ software (Tucker-Davis Technologies). ABR waves were recorded using needle electrodes placed in specific locations: the vertex electrode recorded the reference signal, the mastoid electrode captured the examination signals, and the rump electrode served as the ground. The ABR waveform was determined by averaging 512 responses. Sound intensities ranged from 100 dB SPL to 20 dB SPL, and the auditory threshold was defined as the lowest dB SPL at which an ABR wave was detectable.

### EP measurements.

EP measurements were performed following previously described procedures (52, 53) with minor modifications. Following anesthesia, a ventral tracheotomy was made to secure the airway. The tissue and muscle covering the tympanic bulla were carefully removed, and the bulla was opened to expose the cochlea. A small fenestration was created in the lateral wall of the basal turn using a fine drill. Glass capillary microelectrodes (5–8 MΩ, filled with 150 mM KCl) were mounted on a micromanipulator, and a ground electrode was placed in the abdominal musculature. The recording electrode was then advanced through the spiral ligament and stria vascularis into the scala media. Signals were amplified in current-clamp mode with an Axopatch 200B amplifier and digitized using a Digidata 1440A interface. Continuous voltage traces were acquired in gap-free mode using pClamp 10 (Molecular Devices) at a 1 kHz sampling rate. Data analyses were performed using Clampfit and Microsoft Excel.

### Statistics.

Statistical analyses were conducted with Prism 10 (GraphPad software). Data are presented as the mean ± SD. For comparisons between 2 datasets, an unpaired, 2-tailed *t* test was used if the data followed a normal distribution. For comparisons involving 3 or more datasets, ordinary 1-way or 2-way ANOVA was applied when the data conformed to a normal distribution, whereas the Kruskal-Wallis test was applied for non-normally distributed data. A *P* value of less than 0.05 was considered statistically significant. Sample sizes and additional methodological details are provided in the figure legends.

### Study approval.

All animal experiments were conducted in accordance with animal welfare guidelines approved by the IACUC of the National Taiwan University College of Medicine (approval numbers: 20200082 and 20230090).

### Data availability.

All data associated with this study are included in the article or the supplemental material. Supporting datasets, including quantified values and analysis outputs, are provided in the Supporting Data Values file.

## Author contributions

YHT performed neonatal injections, histology, immunostaining, and EP measurements and wrote the manuscript. PYW performed injections and audiometric tests in juvenile mice. YCC assisted in performing AAV tropism in the ES. CYH performed mouse genotyping. HT and HH provided technical support for the EP measurements. CYH and CCW gave feedback on the manuscript. CCW and YFC designed the study and wrote the manuscript.

## Conflict of interest

This study was partially sponsored by Akouos Inc., a wholly owned subsidiary of Eli Lilly and Company. Akouos provided AAV.Anc80L65 vectors for the research and had no role in study design, data collection, analysis, interpretation, or manuscript preparation. The authors also disclose that a patent application related to this work, “Compositions and Methods for Treating slc26a4-associated Hearing Loss” (US20230201372A1), has been filed.

## Funding support

- National Health Research Institutes (NHRI-EX113-11005NI).
- National Science and Technology Council (NSTC113-2314-B-A49-023-MY3, NSTC114-2314-B-A49-056-MY3).
- Taipei Veterans General Hospital-National Taiwan University Hospital Joint Research Program (VN110-10, VN114-14).
- Taipei Veterans General Hospital (V111C-162, V114C-122).
- Partial funding from Akouos Inc., a wholly owned subsidiary of Eli Lilly and Company.
- AMED-CREST (Multi-sensing: 25gm1510004) grant (to HH).

## Supplementary Material

Supplemental data

Supporting data values

## Version 1 02/17/2026

In-Press Preview

## Version 2 04/15/2026

Electronic publication

## Version 3 04/20/2026

Added graphical abstract
