# Supplementary material for: Postnatal Slc26a4 gene therapy improves hearing and structural integrity in a hereditary hearing loss model
Source: J Clin Invest. 2026 Feb 17;136(8):e193812. doi: 10.1172/JCI193812 (PMC13078887; doi:10.1172/JCI193812)
Supplement: Supplemental data [file jci-136-193812-s007.pdf]

Supplementary materials

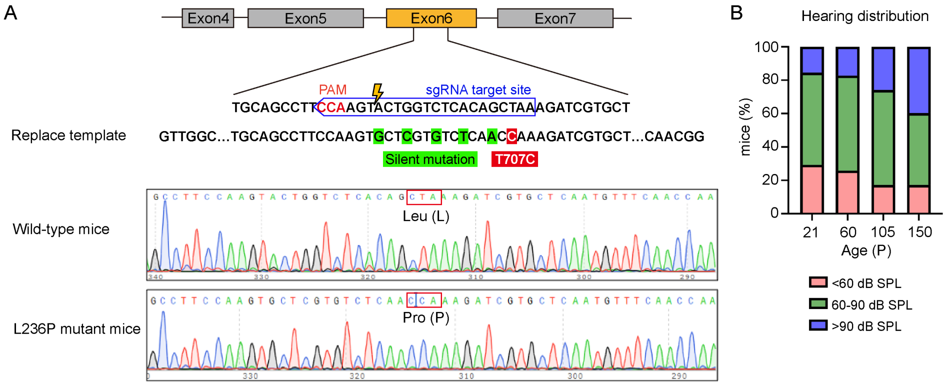

Supplementary Fig. 1. L236P mutant mice generation and hearing distribution. (**A**) Schematic representation of CRISPR/Cas9-mediated generation of the L236P mutant. The designed sgRNA (20 bp) recognizes the protospacer adjacent motif (PAM) sequence to introduce a double-strand break (DSB) at the target site on *Slc26a4* exon 6, along with the template DNA provided for HDR. The template included a silent mutation (green) and the target mutation (red). When the *Slc26a4* c.707 position changed from T to C, it resulted in an amino acid substitution, changing leucine (Leu) to proline (Pro). Sanger sequencing revealed the genomic DNA sequences of WT and *Slc26a4* L236P mutant mice, with the target amino acid mutation from Leu (L) to Pro (P) highlighted in the red box. (**B**) Distribution of hearing levels of L236P mutant mice at P21, P60, P105, and P150. Hearing categories are represented by red (<60 dB SPL), green (60–90 dB SPL), and blue (>90 dB SPL) for each time point.

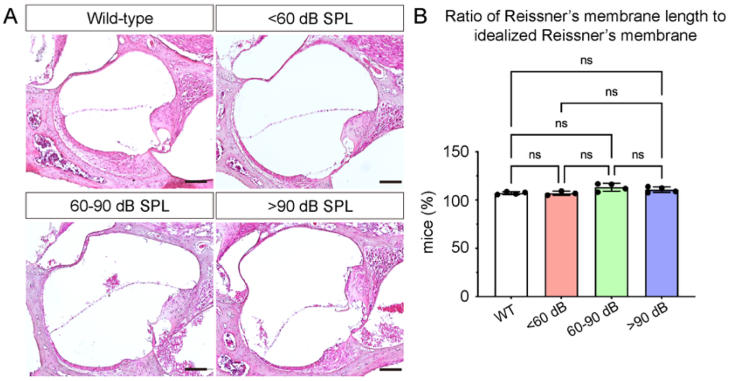

Supplementary Fig. 2. Evaluation of cochlear hydrops in L236P mice at P21.
(**A**) Representative cochlear sections from wild-type FVB controls and the three hearing-loss subgroups of L236P mice at P21. Scale bar =50 µm. (**B**) Quantification of scala media size based on the ratio of the measured Reissner's membrane length to the idealized reference length (Kruskal-Wallis test with Dunn's multiple comparisons test; ns, not statistically significant).

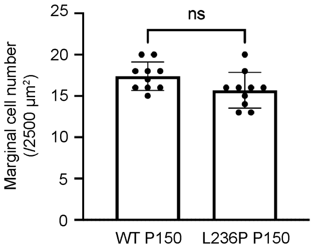

Supplementary Fig. 3. Marginal cell number in L236P mice at P150.
Quantification of marginal cell number in wild-type and L236P mice at P150 (unpaired t-test; $p = 0.07$; n = 10 per group; ns, not statistically significant).

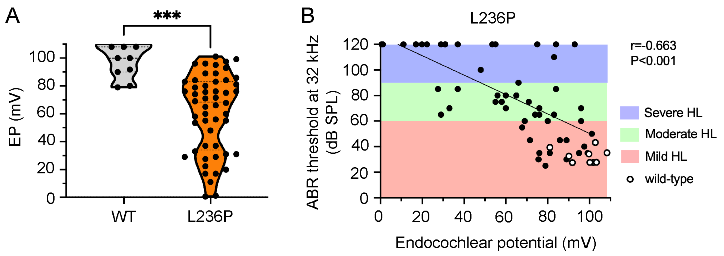

Supplementary Fig. 4. Endocochlear potential of L236P mice at P21.

(**A**) Endocochlear potential (EP) values measured in L236P mice (n = 52) and age-matched wild-type controls (WT, n = 11) at P21. Data are expressed as mean ± SD (***$p < 0.001$, unpaired t-test). (**B**) Correlation between EP and 32 kHz hearing threshold in L236P mice. Spearman's rank correlation analysis revealed a significant negative correlation between EP and 32 kHz hearing threshold (r = -0.663, $p < 0.001$).

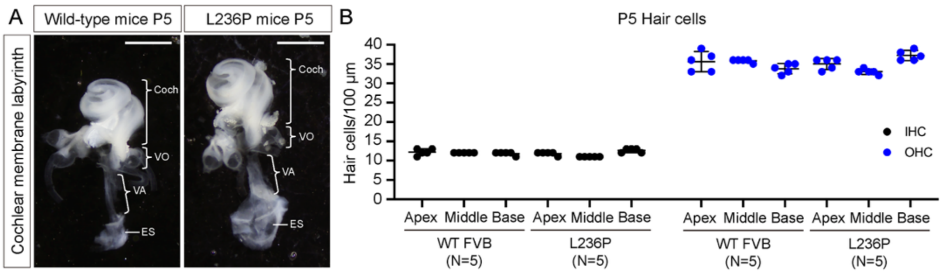

Supplementary Fig. 5. Cochlear morphology of L236P mutant mice at P5.

(**A**) Cochlear membranous labyrinth of wild-type mice and L236P mutant mice at P5, highlighting cochlear duct (Coch), vestibular organs (VO), vestibular aqueduct (VA), and endolymphatic sac (ES). Scale bar, 1 mm. (**B**) Quantification of inner hair cells (IHC, black dots) and outer hair cells (OHC, blue dots) in wild-type and L236P mutant mice at P5. Data are expressed as mean ± SD.

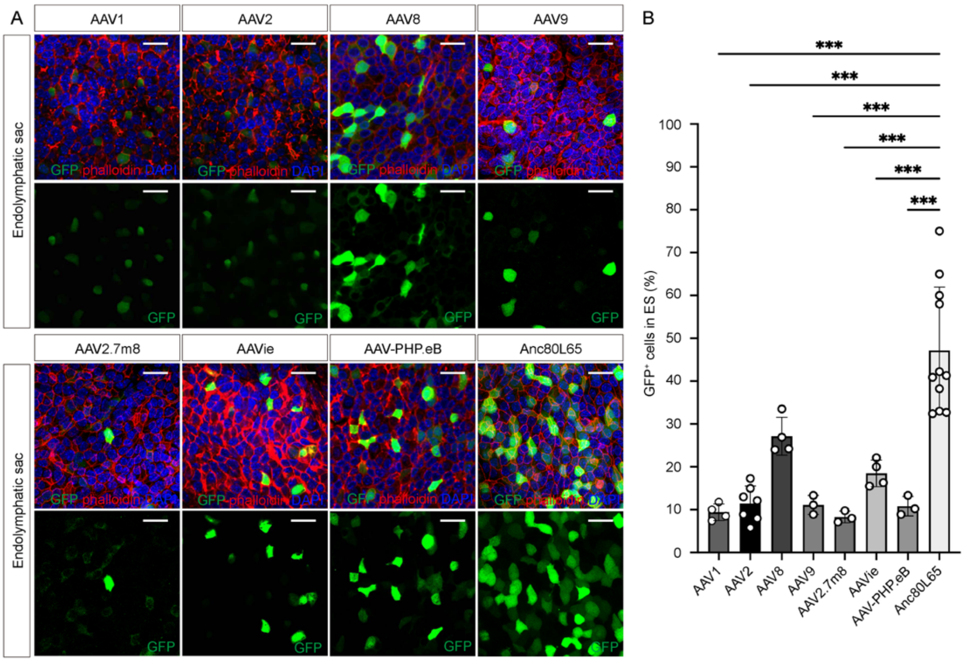

Supplementary Fig. 6. Evaluation of the transduction efficiency of multiple AAV vectors in the endolymphatic sac.

(**A**) Confocal images of the endolymphatic sac cells showing GFP expression in cells transduced by AAV vectors. The green, red, and blue channels represent GFP, phalloidin, and DAPI, respectively. Scale bar =50 µm. (**B**) Quantification of transduction efficiency in the endolymphatic sac for multiple AAV vectors. Data are expressed as mean ± SD (***p < 0.001, Ordinary one-way ANOVA with Tukey's multiple comparisons test).

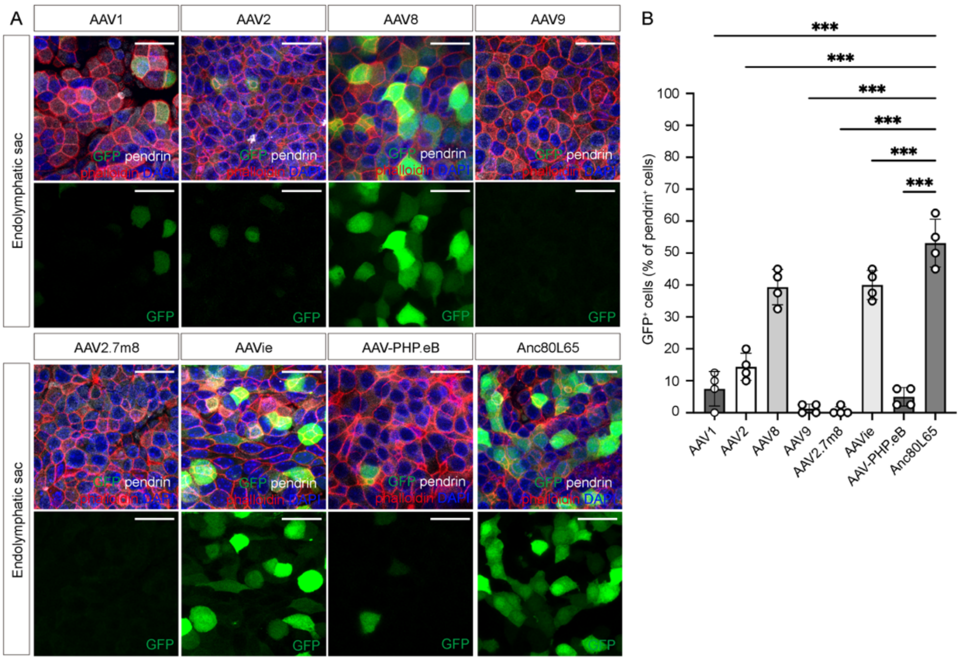

Supplementary Fig. 7. Transduction efficiency of AAV vectors in pendrin-positive cells of the endolymphatic sac.

(**A**) Confocal images of the endolymphatic sac cells showing GFP expression in cells transduced by AAV vectors. Channels: GFP (green), pendrin (gray), phalloidin (red), and DAPI (blue). Scale bar = 20 µm. (**B**) Quantification of GFP transduction efficiency in pendrin-positive cells of the endolymphatic sac for multiple AAV vectors. Data are expressed as mean ± SD (***$p < 0.001$, one-way ANOVA with Tukey's multiple comparisons test).

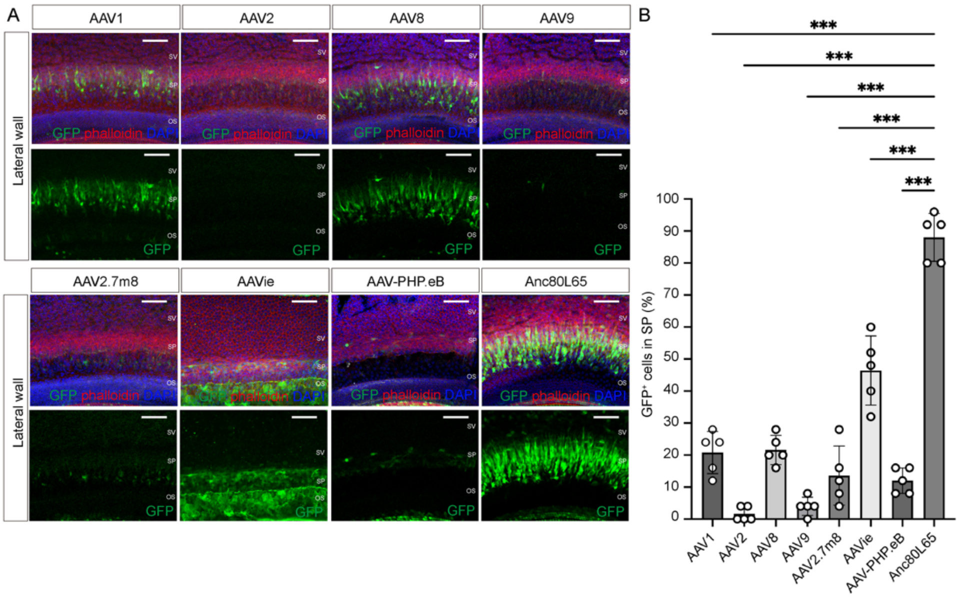

Supplementary Fig. 8. Evaluation of the transduction efficiency of multiple AAV vectors in the spiral prominence.
(**A**) Confocal images of the spiral prominence cells showing GFP expression in cells transduced by AAV vectors. The green, red, and blue channels represent GFP, phalloidin, and DAPI, respectively. Scale bar = 50 µm. SV, stria vascularis; SP, spiral prominence; OS, outer sulcus. (**B**) Quantification of transduction efficiency in the spiral prominence region for multiple AAV vectors. Data are expressed as mean ± SD (***p < 0.001, Ordinary one-way ANOVA with Tukey's multiple comparisons test).

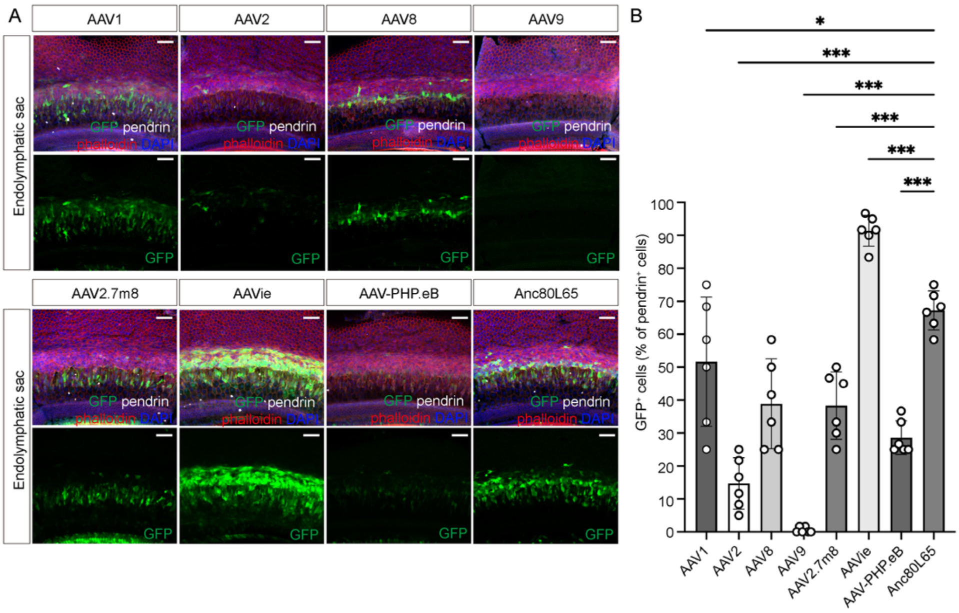

Supplementary Fig. 9. Transduction efficiency of AAV vectors in pendrin-positive cells of the spiral prominence.

(**A**) Confocal images of the spiral prominence cells showing GFP expression in cells transduced by AAV vectors. Channels: GFP (green), phalloidin (red), and DAPI (blue). Scale bar = 50 µm. (**B**) Quantification of GFP transduction efficiency in pendrin-positive cells of the spiral prominence region for multiple AAV vectors. Data are expressed as mean ± SD (**p* = 0.01, ****p* < 0.001, one-way ANOVA with Tukey's multiple comparisons test).

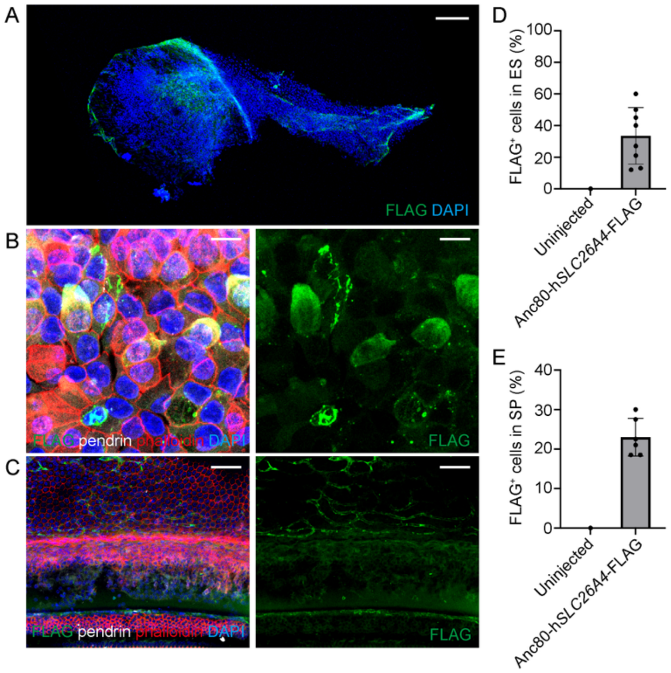

Supplementary Fig. 10. Transduction efficiency of Anc80.h*SLC26A4* vector in the endolymphatic sac and lateral wall cells following juvenile injection.
(**A-B**) The transduction tropism of Anc80.h*SLC26A4* in endolymphatic sac of the wild-type mice. Confocal images under 10X (A) and 63X (B) magnifications. Channels: FLAG (green), pendrin (gray), phalloidin (red), and DAPI (blue). Scale bar 200 µm in A, 10 µm in B. (**C**) The transduction tropism of Anc80.h*SLC26A4* in spiral prominence cells in the lateral wall of the wild-type mice. Channels: FLAG (green), pendrin (gray), phalloidin (red), and DAPI (blue). Scale bar=50 µm. (**D**) Quantification of transduction efficiency in the endolymphatic sac based on images from (B). Data are presented as mean ± SD (n = 4 mice). (**E**) Quantification of transduction efficiency in the spiral prominence based on images from (C). Data are presented as mean ± SD (n = 6 mice).
